# Incremental yield in detection of TB by testing multiple stool specimens from one person

**DOI:** 10.5588/ijtldopen.24.0605

**Published:** 2025-04-09

**Authors:** E. Tiemersma, B. Yenew, G. Diriba, D. Jerene, A. Bedru, P. de Haas

**Affiliations:** ^1^ KNCV Tuberculosis Foundation, The Hague, The Netherlands;; ^2^ Ethiopian Public Health Institute, Addis Ababa, Ethiopia;; ^3^ KNCV Tuberculosis Foundation Ethiopia, Addis Ababa, Ethiopia.

**Keywords:** tuberculosis, diagnostics, children, nucleic acid amplification test, paucibacillary disease, Ethiopia

Dear Editor,

Young children cannot easily produce a spontaneous sputum sample, which hinders bacteriological diagnosis of TB and potentially leads to increased morbidity and mortality.^1^ Following WHO recommendations,^2^ stool-based testing using Xpert MTB/RIF (Ultra) (Xpert) has been successfully introduced in many high TB burden countries,^3^ contributing to increased TB case notifications and bacteriological confirmation rates among children.^4^ Despite this, diagnosing childhood TB remains difficult due primarily to the paucibacillary nature of TB in children. To improve the diagnostic yield, WHO recommended concurrent testing of stool and respiratory specimens for children.^5^ Although testing more than one non-invasive sample was not included in the recommendation,^5^ testing of multiple non-invasive samples might improve TB detection among people who are unable to produce sputum, including children. Applying the logic of testing two or three different sputum samples to improve TB detection by sputum smear microscopy^6^ or Xpert Ultra,^7^ we, therefore, assessed whether testing multiple aliquots from the same or different stool specimens might lead to a significant increase in the bacteriological confirmation of TB.

Details of this study have been published elsewhere.^8^ Briefly, we collected stool from children aged ≤10 years with *Mycobacterium tuberculosis* (MTB) detected using Xpert-Ultra in their respiratory or stool sample,^9^ and adults diagnosed with TB by sputum Xpert in the same health facilities. Stool was collected on three consecutive days within five days after starting anti-TB treatment and transported in cold chain within 24 hours to the national reference laboratory in Addis Ababa, Ethiopia. Each stool specimen was split into aliquots, taking the first aliquot from the opposite of the stool container’s label (‘North’), the second from South, and the third from East or West so that a maximum of nine aliquots were available per participant. All aliquots were immediately processed using the Simple One-Step (SOS) stool test with Xpert Ultra (https://www.kncvtbc.org/uploaded/2021/03/Stoolbox-SOP1.pdf). We collected demographic data, Xpert Ultra results (MTB-positive/negative) of stool and respiratory samples from the participants’ healthcare facilities, and semiquantitative Xpert-Ultra results for all stool aliquots in a preformatted EpiData-file (www.epidata.dk) which was cross-checked by laboratory staff for completeness and correctness. Data were analysed using Stata v18.0/SE (StataCorp, College Station, TX, USA), including participants with results for all nine tested aliquots. We calculated the positivity rate (defined as the number of samples in which Xpert Ultra detected MTB, including trace calls, divided by the total number of samples included in the analysis) of any one, two, and three aliquots tested.^10^ A result was considered ‘positive’ if MTB was detected in any aliquot. Using simple random sampling without replacement with 1,000 repeats, we selected 1–3 aliquots and calculated the mean MTB positivity rate with Wilson confidence intervals. We calculated the incremental yield by dividing the difference between the proportion being MTB-positive after 2 (or 3) aliquots vs. testing 1 (or 2) aliquots by the proportion being MTB-positive when testing 1 (or 2) aliquots.^11^ We stratified by age group (children vs. adults) and bacillary load (paucibacillary TB, i.e. average Xpert Ultra result MTB detected as very low, trace or below trace vs. non-paucibacillary TB, i.e. MTB detected low, medium or high). Caregivers of the children and adult participants provided written informed consent to participate in the study. The study protocol was approved by the Ethical Review Board of the Ethiopian Public Health Institute, Addis Ababa, Ethiopia (EPHI-IRB-234-2020).

We enrolled 48 participants (12 children, 36 adults) with 140 stool specimens and 418 aliquots (100 from children, 318 from adults). Twenty-two aliquots from two adults and three children had been collected >5 days after the start of TB treatment. The complete case analysis included eight children and 33 adults. In 22% of the aliquots collected from patients with paucibacillary TB, Xpert-Ultra did not detect TB, whereas MTB was detected in all aliquots of non-paucibacillary TB patients (*P* < 0.0001) (see Figure). All adults and 6/8 children (75%) had MTB detected in their respiratory sample, vs. 86% (95% confidence interval [CI] 71–94) and 59% (95% CI 28–83) in any one stool aliquot. Their respiratory and stool samples combined were MTB-positive for all children, whereas 74% (40–92) and 83% (49–96) were MTB-positive on two and three aliquots from the same stool, respectively. Our data suggest that 1) the incremental yield of testing two, rather than one, stool aliquots per patient is 20–25% among persons with paucibacillary TB and children; 2) the additional yield of adding a third aliquot is consistently lower than the incremental yield of adding a second aliquot for testing; 3) there is no difference in incremental yield of testing aliquots from the same stool specimen compared to testing aliquots from different specimens (Table); and 4) the rate of non-determinate results is 10% (Figure).

**Figure.**
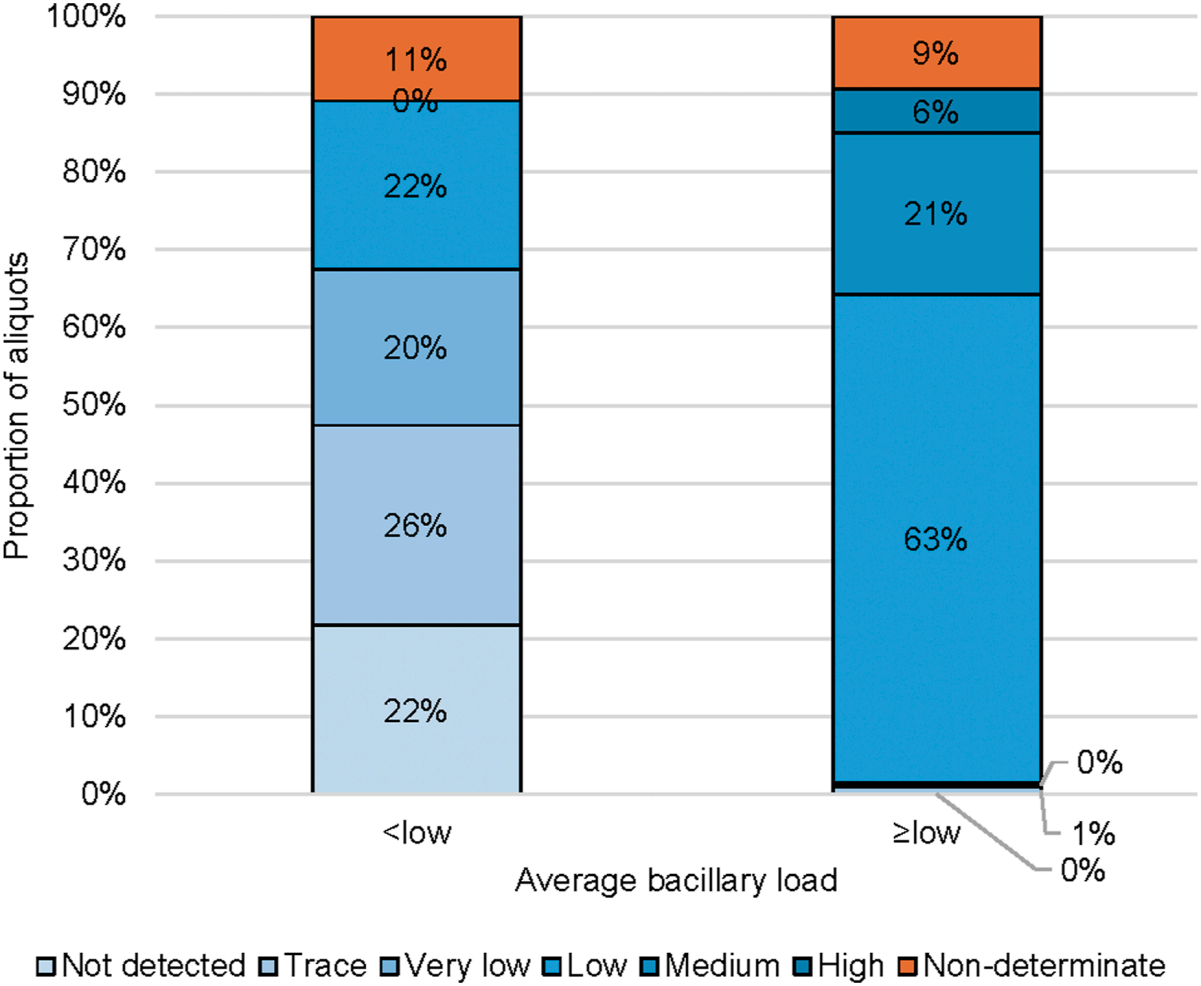
Result per aliquot by semiquantitative Xpert Ultra result averaged over all aliquots of a patient, as either <low (<trace/trace/very low, i.e., paucibacillary TB) or ≥low (low/medium/high, i.e., non-paucibacillary TB).

**Table.** Proportion of samples with a *Mycobacterium tuberculosis* (MTB) positive Xpert Ultra result when testing one, two or three stool samples from the same patient and different stool specimens by sputum result.

Type of aliquots included	Any 1 aliquot from any stool specimen	Any 2 aliquots from 1 random stool specimen	Any aliquot from 2 different stool specimens	3 aliquots from 1 random stool specimen	3 aliquots from 3 different stool specimens
Overall (*n* = 41)					
Number of stool specimens included	41	41	82	41	123
Number of aliquots included	41	82	82	123	123
Mean MTB+, % (95% CI)	81 (67–90)	92 (79–97)	91 (78–96)	97 (86–99)	94 (84–99)
Incremental yield of adding 1 aliquot, %	NA	14	12	5	3
Incremental yield of adding 2 aliquots, %	NA	NA	NA	20	16
By age group, years					
Children <10 years (*n* = 8)					
Number of stool specimens	8	8	16	8	24
Number of aliquots	8	16	16	24	24
Mean % MTB+ (95% CI)	59 (28–83)	74 (40–92)	71 (38–91)	83 (49–96)	79 (41–93)
Incremental yield of adding 1 aliquot, %	NA	25	20	12	11
Incremental yield of adding 2 aliquots, %	NA	NA	NA	41	34
Adults ≥18 years (*n* = 33)					
Number of stool specimens	33	33	66	33	99
Number of aliquots	33	66	66	99	99
Mean % MTB+ (95% CI)	86 (71–94)	96 (84–99)	95 (82–99)	100 (90–100)	98 (85–99)
Incremental yield of adding 1 aliquot, %	NA	12	10	4	3
Incremental yield of adding 2 aliquots, %	NA	NA	NA	16	14
By average bacterial load					
Paucibacillary TB (<trace/trace/very low) (*n* = 15)					
Number of stool specimens	15	15	30	15	45
Number of aliquots	15	30	30	45	45
Mean % MTB+ (95% CI)	67 (44–84)	82 (59–93)	84 (62–94)	83 (62–94)	87 (67–97)
Incremental yield of adding 1 aliquot, %	NA	22	25	1	4
Incremental yield of adding 2 aliquots, %	NA	NA	NA	24	30
Non-paucibacillary TB (low/medium/high) (*n* = 26)					
Number of stool specimens	26	26	52	26	78
Number of aliquots	26	52	52	78	78
Mean % MTB+ (95% CI)	91 (74–97)	98 (82–100)	98 (83–100)	98 (83–100)	99 (86–100)
Incremental yield of adding 1 aliquot, %	NA	8	8	0	1
Incremental yield of adding 2 aliquots, %	NA	NA	NA	8	9

CI = confidence interval; NA = not applicable.

Testing more than one aliquot of stool may especially benefit those with paucibacillary TB, including children. The initial sputum or gastric aspirate (GA) Xpert-Ultra result and stool results suggest that testing one respiratory plus one stool sample has a higher yield than testing two aliquots of stool, corresponding with the higher sensitivity of Xpert-Ultra on a respiratory compared to a stool sample.^12^ However, testing an additional aliquot of an available stool specimen in a primary healthcare facility could save parents and the health system referral costs and prevent delayed diagnosis and additional (invasive) tests for some children.^1^ An increased diagnostic yield, similar to testing two GA samples, was found when combining nasopharyngeal aspirates (NPA) with GA, stool or urine samples.^13,14^ However, for children, collection of NPA causes stress, anxiety and pain.^15^ Truly non-invasive samples, such as stool or urine (and possibly tongue swabs), are to be preferred. A limiting factor may be the availability of cartridges, as many countries face difficulties in securing Xpert cartridges.

Our study is limited by its sample size, resulting in wide confidence intervals and prohibiting drawing firm conclusions on the added value of testing more than one aliquot of stool for people with paucibacillary TB. Nevertheless, we believe that there is value in testing two samples for persons with presumptive paucibacillary TB, including young children. Priority should be placed on non-invasive samples with a high sensitivity for TB detection using molecular, WHO-recommended rapid diagnostics.

Further research is needed to investigate if the sensitivity of (stool) Xpert testing can be improved, (e.g. by using transport media such as Primestore Molecular Transport Medium (Longhorn Vaccines and Diagnostics, LLC, Bethesda, Maryland, USA) or by applying other combinations of non-invasive samples), and if tests with lower cost than Xpert can be applied.
